# The Lipidic and Volatile Components of Coffee Pods and Capsules Packaged in an Alternative Multilayer Film

**DOI:** 10.3390/foods13050759

**Published:** 2024-02-29

**Authors:** Giulia Basile, Lucia De Luca, Martina Calabrese, Gianfranco Lambiase, Fabiana Pizzolongo, Raffaele Romano

**Affiliations:** 1 Department of Agricultural Sciences, University of Naples Federico II, Via Università 100, Portici, 80055 Napoli, Italy; giulia.basile@unina.it (G.B.); lucia.deluca@unina.it (L.D.L.); martina.calabrese@unina.it (M.C.); raffaele.romano@unina.it (R.R.); 2 Flessofab s.r.l., Montemiletto, 83038 Avellino, Italy; lambiase@flessofab.it

**Keywords:** oxidation, peroxides, storage, polyethylene terephthalate (PET), volatile organic components

## Abstract

Coffee pods and capsules require packaging that guarantees the optimal coffee preservation. The chemical composition of coffee can undergo quality decay phenomena during storage, especially in terms of lipidic and volatile components. Amongst coffee packaging, aluminum multilayer materials are particularly widely diffused. However, aluminum is a negative component because it is not recoverable in a mixed plastic structure and its specific weight gives significant weight to packaging. In this study, a multilayer film with a reduced content of aluminum was used to package coffe pods and capsules and compared to a standard film with an aluminum layer. Their influence on the peroxides and volatile organic compounds of two coffee blends, 100% *Coffea arabica* L., 50% *Coffea arabica* L., and 50% *Coffea canephora* var. *robusta* L., were studied during their 180-day shelf life. The predominant volatile organic compounds detected belonged to the class of furans and pyrazines. Both packaging materials used for both coffee blends in the pods and capsules showed no significant differences during storage. Thus, the alternative packaging with less aluminum had the same performance as the standard with the advantage of being more sustainable, reducing the packaging weight, with benefits for transportation, and preserving the coffee aroma during the shelf life.

## 1. Introduction

Coffee pods and capsules are single-serving products of roasted and ground coffee. In capsules, the coffee is pressed and placed in a generally cylindrical plastic or aluminum container sealed under vacuum [1]. In pods, the coffee is pressed and placed in round or flat packages made with paper. Their use is convenient for consumers because it enables them to prepare a relatively high-quality coffee product in a short period of time, with minimal water and energy use. The growing demand for high-quality coffee pods and capsules requires packaging that guarantees the optimal coffee preservation, since coffee is a susceptible matrix of chemical changes during its storage time [2,3]. Their shelf life is usually about 18 months or more [4] and is correlated with the packaging materials. Coffee pods and capsules are sealed in a high-barrier metalized package flushed with an inert gas (typically N_2_) prior to sealing [5].

The disadvantage of pods and capsules is that they are not very sustainable for the environment due to the aluminum foil contained in the packaging that has to be discarded as waste. Moreover, aluminum foil is not recoverable in a mixed plastic structure and its specific weight gives a significant weight to packaging. In recent years, the food packaging industry has been moving toward solutions that best meet the requirements of circularity and sustainability and, therefore, must also pay attention to environmental needs [6]. The first step towards sustainability is layer reduction of the non-recyclable material. Traditional flexible film for coffee consists of multilayer flexible packaging made of polyethylene terephthalate (polyester)-aluminum-polyethylene (PET-AL-PE). PET is used for its distinct mechanical strength and excellent printability, polyethylene (PE) has excellent sealing properties, and the aluminum layer protects the product from the light component and provides a barrier to the insufflated gas in the package [2,7]. There are, however, elements that make aluminum a negative component, because it has high costs and a low environmental performance. Moreover, its specific weight gives a significant weight to packaging that is a problem for transportation. Therefore, replacing or reducing the aluminum foil laminate in multilayer films is recommended from an environmental point of view. In fact, Bayus et al. [8], found that metallized polymer laminates have less of an environmental impact than aluminum foil laminate. They also found that metallized polyethylene terephthalate (MPET) layers have an excellent performance in oxygen transmission rate, that is, the property of utmost importance in preserving food freshness. Therefore, in this work, we considered a multilayer film containing MPET (alternative) in place of aluminum foil to package coffee. Its reduced weight could lead to a benefit in terms of logistics and transportation. In fact, with the same transport, there is the possibility to deliver more products. As reported by Liccardello [9], packaging weight reduction is a potential strategy for overall impact reduction. Furthermore, packaging lightweighting represents the easiest and most accesible measure for sustainability improvement in food packaging [10].

The two commercially relevant species of the *Coffea* genus, *Coffea arabica* L. (commonly known as Arabica) and *Coffea canephora* var. *robusta* L. (commonly known as Robusta), were used inside the pods and capsules. They differ considerably in price, quality, and sensory properties. Therefore, blends of the varieties are produced to obtain the preferred flavor. Arabica is more expensive than Robusta and this is an economic incentive to illicitly replace Arabica with Robusta. Moreover, Arabica represented 57.4% of world production in 2023/2024, while Robusta had a 42.6% market share [11]. Arabica has a slightly sour taste with a stronger aroma and balanced flavor that is favored in international markets, and it is considered to be mild coffee. Meanwhile, Robusta has a bitter taste with a weaker aroma, but its body (thickness in the mouth) is pleasant and it is consumed in emerging and developed markets as a mixed product [12,13].

Coffee is a fairly stable product, but there are physical and chemical changes that occur during storage that could affect its quality [14,15]. Coffee contains a high percentage of unsaturated fatty acids and a low moisture content, and is, therefore, a product susceptible to lipid oxidation and rancidity during storage. In fact, roasted coffee loses the aroma and flavor of ’fresh coffee’ due to some lipid oxidation and the degradation of some compounds inherent to the typical aroma [16]. Becoming greatly odorless and flavorless is the consequence of the formation of volatile compounds with oxygen [17]. The volatile organic compounds (VOCs) in coffee can be divided into different classes, including (in order of abundance) furans, pyrazines, ketones, pyrroles, phenols, hydrocarbons, acids and anhydrides, aldehydes, esters, alcohols, sulfur compounds, and others [18]. Furans exhibit malt and sweet roast aromas with higher sensory thresholds than other VOCs, as reported by Bellumori et al. [19]. Furans are also intermediates for other key compounds like furfurylthiol (roasty) or combined acetates (sweet and fruity). Pyrazines and sulfur-containing compounds are considered as the most significant to coffee flavor [20]. Pyrazines are molecules that derive from coffee roasting and are formed by reactions between carbohydrates and α-amino acids [21]. Ketones and aldehydes are good indicators of coffee quality in the cup, because they depend on the roasting process. By increasing the time and temperature of the roasting process, the pleasant aroma given by pyrazines, aldehydes, and ketones turns into bitter and sour notes [22]. The amount of the coffee aroma compounds decreases steadily because they are highly volatile or labile. Therefore, the use of packaging films with good barrier properties, eliminating oxygen inside the package during the process of coffee packaging, helps to prevent or slow down the oxidative degradation of coffee aroma, subsequently reducing the loss of freshness [23]. 

Several studies have evaluated trends in coffee quality during storage. Glöss et al. [24] evaluated the dimethyl disulfide/methanethiol and 2-butanone/methanethiol ratio as freshness index for single-serve capsules packed in various packaging materials. Kreuml et al. [17] showed the sensorial quality of roasted coffee beans packed under vacuum conditions in commercially available packages and stored at ambient temperature for 18 months of decay after 9 months of storage. Giulia et al. [25] investigated the capability of different coffee capsules, packed with a compostable packaging or packaging based on polypropylene and aluminum, to maintain flavor quality during storage in stressful conditions.

The aim of this research was to compare the physical and chemical properties of coffee pods and capsules packaged in two different films (MPET and PET-AL-PE used as control) and stored at 25 °C and 40 °C for 180 days. Oxygen in the headspace, peroxide value, and volatile organic components were evaluated. The acceptability test was also performed.

## 2. Materials and Methods

### 2.1. Chemicals

All solvents and reagents used for the experiments were purchased from Sigma–Aldrich Co. (Milano, Italy).

### 2.2. Samples

Samples of pods containing 8.5 g of 100% *Coffea arabica* were provided by Kimbo Spa (Naples, Italy). Samples of capsules containing 7.3 g of 50% *Coffea arabica* and 50% *Coffea canephora* var. *robusta* were provided by Barbaro Srl (Naples, Italy). Both types of coffee had a dark roasting degree obtained at 230 °C for 12 min.

The pods were made of paper and the capsules were cylindrical plastic containers.

Two types of flexible multilayer films were used to package the pods and capsules in a protective atmosphere (100% N_2_): aluminum film used as standard (STD) and metallized film used as an alternative (ALT). The pods were individually packaged, while the capsules were in packs of 10.

The composition of the STD film was:

- Polyethylene terephthalate at 12 microns, aluminum at 8 microns, and polyethylene at 60 microns (PET-AL-PE) with an Oxygen Transmission Rate (OTR) of <1.0 and 99 g/m^2^ grammage.

In the ALT film, the aluminum layer was replaced with Metallized Polyethylene Terephthalate (MPET) at 12 microns (aluminum oxide < 1 micron, PET 11 microns) and the composition was:

- Polyethylene terephthalate at 12 microns, Metallized Polyethylene Terephthalate at 12 microns, and polyethylene of 55 microns thick (PET-MPET-PE) with an OTR of <1.5 and 90 g/m^2^ grammage.

Considering the grammage (99 and 90 g/m^2^ in PET-AL-PE and MPET, respectively), a 10% reduction was achieved in the alternative packaging. 

To obtain the final packaging, the polyester was printed by gravure technology using polyurethane-nitro inks and laminated according to solvent-free technology using a two-component polyurethane adhesive.

The samples were stored at 25 °C and 40 °C for 180 days, and at 0, 30, 60, 90, 120, 150, and 180 days, the percentage of oxygen and number of peroxides were evaluated, while at days 0, 90, and 180, the moisture, VOCs and the acceptability (this letter only at 25 °C) were assessed. 

### 2.3. Evaluation of Oxygen Percentage during Shelf Life

The measurement of the percentage of oxygen (%) in the packaging headspace was conducted with a Witt Oxybaby 4.0 analyzer during storage tests, before being opened.

### 2.4. Moisture Content

The moisture content of the coffee powder was determined by drying the samples for 24 h at 105 °C. The results are expressed as a weight/weight percentages of water (% *w*/*w*).

### 2.5. Fat Extraction

Fat extraction from the coffee was performed according to Cong et al. [26], with some modifications. Briefly, approximately 20 g of coffee was extracted with 100 mL of n-hexane in the first extraction cycle, and the extraction was repeated 2 more times with another 50 mL of n-hexane. The mixture was sonicated in an ultrasonic bath for 50 min. At the end of sonication, the mixture was centrifuged at 6500 rpm for 10 min. The organic phase was filtered, dried at 40 °C using a Rotavapor Labourota4000-Efficient instrument (Heidolph Instrument, Schwabach, Germany), and stored at −18 °C until the analysis. The fat extraction yield is expressed as g of oil extracted/100 g of coffee. 

### 2.6. Peroxide Value

The number of peroxides was determined as reported by Romano et al. [27]. Briefly, 1 g of fat extract was added to 10 mL of an acetic acid–chloroform mixture in the ratio (3:2 *v*/*v*). Subsequently, 0.1 mL of a saturated KI solution was added, and finally stirred on a magnetic plate and allowed to react in the dark for about 5 min. After that, 15 mL of water was added to stop the reaction and the solution was titrated, with Na_2_S_2_SO_3_ (0.001 N) in the presence of starch solder as an indicator. The value of the peroxide number is expressed as milliequivalents of oxygen per kg oil (meqO_2_/Kg oil).

### 2.7. Volatile Organic Compounds

The analysis of volatile organic compounds (VOCs) was performed using the solid-phase microextraction technique (SPME) coupled with gas chromatography according to Bertrand et al. [28], with some modifications. Briefly, 2 g of coffee powder was weighed in a 20 mL vial for headspace analysis. The vial was kept at 60 °C for 5 min and, subsequently, a divinylbenzene/carboxen/polydimethylsiloxane (DVB/CAR/PDMS) fiber was introduced into the vial and kept at 60 °C for 7 min. The fiber was inserted in a gas chromatograph injector, where thermal desorption of the analytes was performed at 240 °C for 4 min in splitless mode. A 6890N GC system equipped with a 5973 mass detector was used. The VOCs were separated on an HP-5MS capillary column (30 m × 0.25 mm ID × 0.25 µm) of 5% diphenyl 95% dimethylpolysiloxane. The column oven temperature was increased from 60 °C to 240 °C at 3 °C min^−1^. Helium was used as a carrier gas at a flow rate of 1 mL min^−1^. The ionizing electron energy was 70 eV, and the mass-to-charge ratios were scanned over the range from 40 to 450 amu in full-scan acquisition mode. The injection and ion source temperatures were 250 and 230 °C. The compounds were identified using the NIST (National Institute of Standards and Technology) Atomic Spectra Database version 2.0 and verified for retention indices. The relative content of VOCs was calculated based on peak area ratios and is expressed in terms of percentage (%).

### 2.8. Sensory Evaluation

To test the overall acceptability of the product, a sensory analysis was conducted using a semi-structured hedonic scale [29]. A 42-member panel of untrained consumers with some experience in the sensory evaluation of coffee was recruited. The panellists were 18 women between 17 and 60 years old and 24 men between 19 and 64 years old. The coffee pods and capsules were introduced into a Mokona machine (Bialetti Industri, S.p.a, Coccaglio—BS, Italy) to produce the coffee to drink. Approximately 30 mL of each coffee sample was served in a 55 mL paper-based coffee cup, labelled with a random 3-digit code and served individually to the panelists in random order. All the samples were presented in duplicate with different sample orders. The samples were presented at 70 °C and no condiments were allowed to be added. The tests were performed in an isolated room with good illumination and natural ventilation in groups of 7 subjects at a time. The panelists rinsed their mouths with still water between samples. Overall acceptability was evaluated. Each panelist received a form sheet with a nine-point hedonic scale anchored with “Like Extremely” and “Dislike Extremely” at either end, with a neutral point of “Neither Like nor Dislike”.

### 2.9. Statistical Analysis

All experiments were performed in triplicate, and the results are expressed as the mean values (±standard deviations) of the three replicates. The data were submitted to a one-way analysis of variance (ANOVA) and Tukey’s multiple-range test (*p* ≤ 0.05) using XLSTAT 2023 software (Addinsoft, New York, NY, USA).

## 3. Results

### 3.1. Percentage of Oxygen in the Headspace during Shelf Life

Coffee is a dried product, and it is resistant to spoilage by any microorganisms. But its lipidic components can cause oxidization and may lead to rancidity as well. To avoid this process, the percentage of oxygen is minimized [30]. 

**Figure 1:**
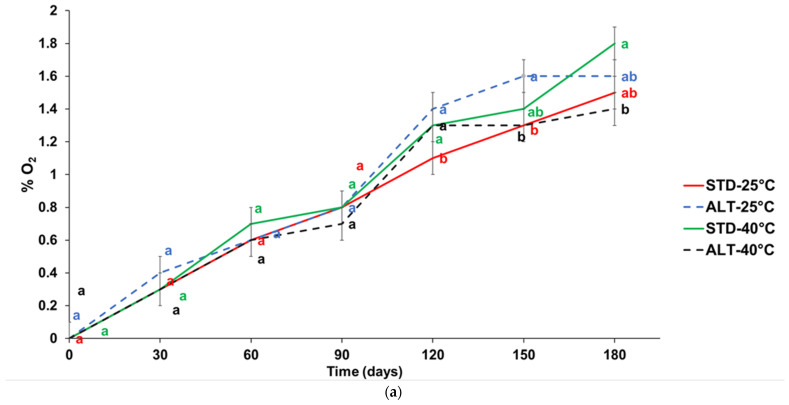

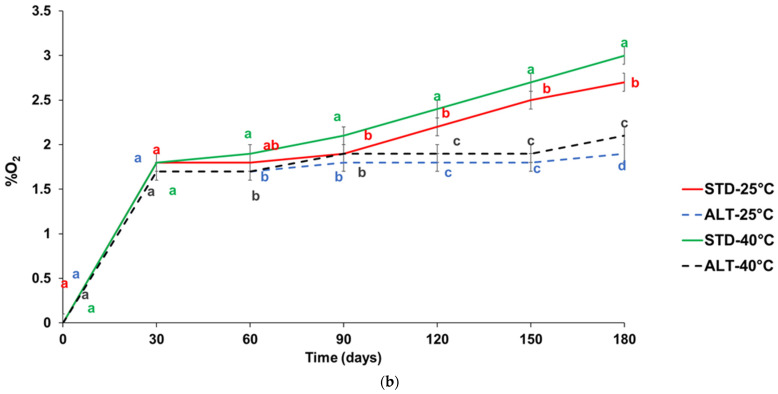
(**a**) Percentage of oxygen in the headspace of coffee pods (100% *Arabica* variety) packaged with standard (STD) and alternative (ALT) multilayer film and stored at 25 and 40 °C for 180 days. ^a–b^ Different letters at the same time indicate statistically significant differences (*p* < 0.05); (**b**) percentage of oxygen in the headspace of coffee capsules (50% Arabica and 50% Robusta varieties) packaged with standard (STD) and alternative (ALT) multilayer film and stored at 25 and 40 °C for 180 days. ^a–d^ Different letters at the same time indicate statistically significant differences (*p* < 0.05).

In Figure 1a,b, the percentages of oxygen in the headspace of the coffee pods and capsules are reported, respectively, packaged with the standard (STD) and alternative (ALT) multilayer films and stored at 25 and 40 °C for 180 days. 

In the pods, the % oxygen after 30 days was approximately 0.4% in both multilayer films and at both storage temperatures, while in the capsules, it was approximately 1.7%. These values slowly increased to 1.4–1.8% in the pods and 1.9–3% in the capsules after 180 days of storage, because the amount of gas permeating was directly proportional to time. The percentage of oxygen in the pods was lower than that in the capsules because the volume of gas that permeated in the time was directly proportional to the surface area [31]. 

At both temperatures, an increase in the percentage of O_2_ in the pods (Figure 1a) for both packages was shown; however, in pod storage at 40 °C, this increase was lower in the alternative packaging than in the standard. 

The percentage of oxygen in the capsules packaged with ALT (Figure 1b) was significantly lower than those in the STD film from 120th day of storage, indicating an excellent oxygen barrier performance in the ALT film. As reported by Baggenstoss et al. [32], ground, roasted coffee packaged in sealed portions showed a rapid decrease in levels of 2-furfurylthiol, an important compound of coffee aroma [33], at oxygen levels equal to or greater than 5%, while at an oxygen concentration of approximatively 2%, this decrease was slowed. Using packaging with good oxygen barrier properties helps to prevent or slow down the oxidative degradation of coffee aroma, subsequently reducing the loss of freshness properties [23]. In capsules, a temperature effect for both types of packaging was shown, in fact, at 40 °C, the O_2_ concentration was higher than in those stored at 25 °C. However, at both storage temperatures, the capsules with alternative packaging showed a lower percentage of O_2_ during storage than those in the standard.

### 3.2. Moisture Content

Coffee is a highly hygroscopic matrix [34], and moisture is a factor in establishing the shelf life of dried products. In Table 1, the moisture contents of the coffee pods and capsules during storage are reported. As reported by Augustini and Yusya [35], during the storage of the pods and capsules, there was an increase in moisture. For the pods at 25 °C, there was an increase in the moisture value of up to 2.30 percent for the alternative packaging and 2.15 percent for the standard packaging. At 40 °C, there was a slightly larger increase (2.73% for alternative packaging and 2.83% for standard packaging). No statistical differences were found among the different packaging, and temperature did not statistically affect the moisture value either. For capsules, the behavior was the same. At 180 days at 25 °C, the standard packaging had a value of 2.59%, while the alternative had a value of 2.70%. The temperature of 40 °C resulted in slightly higher but not statistically different moisture values. During coffee storage, there was an increase in moisture due to transmission from the surrounding environment. As reported by Agustuni and Yusya [34], most international standards for the quality of roasted ground coffee state that the moisture content should not exceed 5.0% at the time of packaging.

### 3.3. Fat Yield

The fat yield of the pods (100% *C. arabica*) was 10.85%, while in the capsules (blend 50% *C. arabica* and 50% *C. canephora* var. *robusta*), it was 9.48%. These values are similar to those reported by Rubayiza and Meurens [22], who found 16.8% fat in 100% *C. arabica* and 11.5% in *C. canephora* var. *robusta*.

### 3.4. Peroxide Values

Peroxides and hydroperoxides, the primary products of fat oxidation, can be used as an oxidative index for the early stages of lipid oxidation. A good oxidative stability is usually accompanied by a slower increase in peroxide values [26].

**Figure 2:**
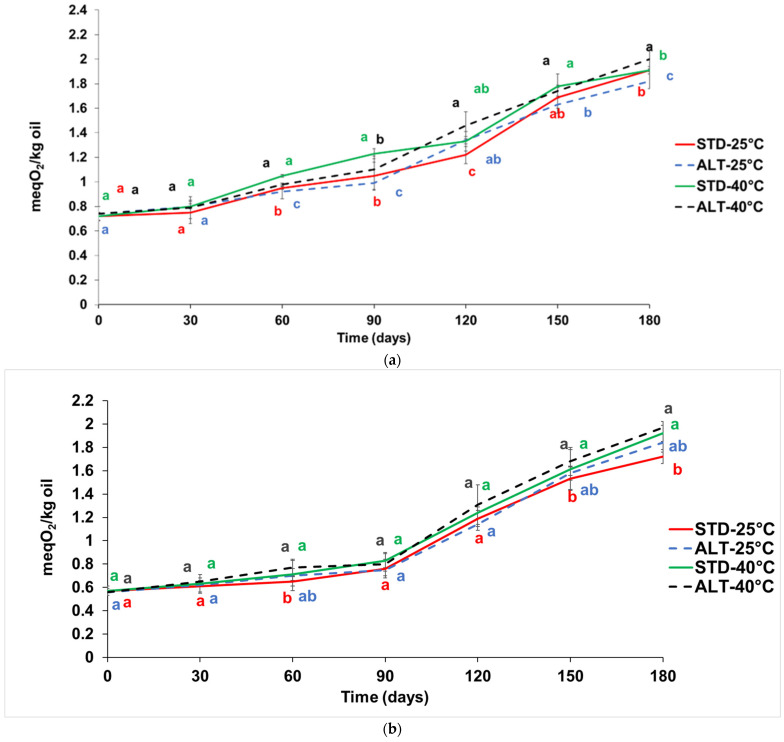
(**a**) Peroxides value (meq O_2_/Kg oil) in coffee pods (100% *Arabica* variety) packaged with standard (STD) and alternative (ALT) multilayer films and stored at 25 and 40 °C for 180 days. ^a–c^ Different letters at the same time indicate statistically significant differences (*p* < 0.05); (**b**) peroxides value (meq O_2_/Kg oil) in coffee capsules (50% Arabica and 50% Robusta varieties) packaged with standard (STD) and alternative (ALT) multilayer film and stored at 25 and 40 °C for 180 days. ^a–b^ Different letters at the same time indicate statistically significant differences (*p* < 0.05).

In Figure 2a,b, the trend of peroxide values during storage is shown in the coffee pods and capsules, respectively. A small increasing trend was observed in both samples.

In the coffee pods, the peroxide values increased from 0.70 to 1.91 meqO_2_/Kg of fat in the standard and to 1.82 meqO_2_/Kg of fat in the alternative packaging during storage at 25 °C. The initial value was similar to Hong et al. [36], who reported a value of 0.97 meq/kg oil in green coffee beans.

In the coffee capsules, the peroxide values increased from 0.59 to 1.72 meqO_2_/Kg of fat in the standard and to 1.84 meqO_2_/Kg of fat in the alternative packaging, respectively. Turatti [37] reported that the value of peroxides until 2.41 meqO_2_/Kg of oil was not correlated with the rancid sensory indicator. The peroxide values during storage in both packaging and at both temperatures were lower than those reported by Getachew and Chu [38], who found a sharp increase after 12 weeks of storage at 45 °C up to 7.21 meq O_2_/Kg fat. Frascareli et al. [39] showed similar values and trends during the storage of coffee oil at 25 °C. Anese et al. [40] showed a peroxide value of less than 2.00 meq O_2_/Kg fat during storage at 30 °C. Coffee has a high antioxidant power due to its phenolic compounds and products of the Maillard reaction [41], which can help to prevent oxidation and thus the formation of peroxides.

Comparing the standard and alternative packaging in samples stored at the same temperature and time, no statistically significant differences were found in the peroxide values, indicating a good performance of the latter.

### 3.5. Volatile Organic Compounds

Coffee volatile compounds include several chemical classes, like hydrocarbons, alcohols, aldehydes, ketones, carboxylic acids, esters, pyrazines, pyrroles, pyridines, other bases (e.g., quinoxalines and indoles), sulfur compounds, furans, furanones, phenols, and oxazoles, among others [18].

In Table 2 and Table 3, the volatile organic compounds (VOCs) contents found in the coffee pod and capsule samples stored at 25 °C and 40 °C, respectively, are reported. In both samples, the most abundant classes of VOCs were furans and pyrazines (>27%). 

In the pods (Table 2), furans were present in the highest content in the coffee stored in the alternative packaging at all analyzed time and temperatures, with an increase during the shelf life found, while in the capsules (Table 3), the furan content increased during the shelf life compared to time 0, but only at 90 days and 180 days was a higher content in ALT compared to STD found.

Pérez-Martínez et al. [42] showed no significant differences in total furans content in coffee brews storage at 25 °C and 4 °C, but 2-methylfuran, 3-methylfuran, and 2,5-dimethylfuran decreased in samples stored at 25 °C, while other furans such as 2-vinylfuran, 2-vinyl-5-methylfuran, 2-furfuryl acetate, and 2-furfurylfuran, showed, in general, a significant decrease at both storage temperatures. Roasted coffee beans contain furan in the highest concentration compared with other food, and in coffee, the concentration depends on roasting temperature, roasting time, particle size of coffee ground, and type of preparation [43].

Pyrazines during shelf life decreased and were most present in the standard packaging compared to the alternative in both capsules and pods. Among this class, alkylpyrazines are considered to be key aroma components of coffee brew and these compounds contain the lowest odor threshold, so they contribute to developing the coffee aroma [21]. 

Aldehydes could be derived from the oxidative degradation of amino acids during their interactions with sugars at high temperatures or polyphenols in the presence of polyphenol oxidase [21]. Furthermore, aldehydes are secondary compounds of oxidation and influence coffee acceptability. It is interesting to note that hexanal was not found, indicating a good preservation of the samples in all the packaging films used. This compound, in fact, is responsible for rancid flavor and its presence in coffee during storage could be used as a marker of coffee staling or freshness index [44]. The predominant aldehyde detected was 2-butenal. It increased in the coffee pods during the storage period and its concentration was higher in the standard packaging compared to the alternative packaging after 180 days of storage both at 25 and 40 °C. Furthermore, it was also found in the coffee capsules after 180 days of storage at both temperatures, with a higher increase in the coffee packaged in the standard packaging compared to the alternative packaging. This compound could be derived from the oxidation of ω-3 polyunsaturated fatty acids (PUFAs) and subsequent decomposition of hydroperoxides [45]. 

Among ketones, 3-hydroxy-2-butanone was found. This compound, derived from the sugar decomposition reaction, occurs in the Maillard reaction, as well as in caramelization [46]. Among phenols, guaiacol was the most predominant, and gives smoky and spicy tones [1]. 

In this study, two volatile organic compounds were selected to monitor the evolution of the coffee shelf life: acetic acid and 5-methylfurfural. These molecules were identified by Korhonová et al. [47] in their study on the major VOCs present in coffee.

Acetic acid tends to form during roasting processes through the Maillard reaction between reducing sugars and amino acids and sugars, and its concentration is influenced by roasting time, with an increase when time is increased [48]. Acetic acid is associated with sourness, as well as rancidity, astringency, and bitterness [49,50,51]. In coffee brews, it has been reported that its concentration increases throughout storage time and is positively influenced by storage temperature [42]. Acetic acid increased during the shelf life both in the pods and capsules (Table 2 and Table 3). In pods, at the 180th day at 25 °C, the values of acetic acid were 8.50% and 8.24% for the alternative and standard packaging, respectively, while at 40 °C, these were 10.11% for the standard packaging and 10.27% for the alternative packaging. At 25 °C, there was an increase of 1.13% for the standard packaging and 1.39% for the alternative packaging, but both films showed no statistically significant differences at the end of the study. For the capsules, at both 25 °C and 40 °C, the alternative packaging showed higher values of acetic acid: 5.84% vs. 3.71% at 25 °C and 8.83% vs. 3.71% at 40 °C. Also, Cincotta et al. [2] reported an increase in acetic acid in coffee packaged in capsules. Probably, lipids may be involved, because a continuous decrease in triglycerides is associated with an increase in free fatty acids during shelf life [52]. Furthermore, the increase in acetic acid can be attributed to the hydrolysis of esters and, consequently, an increase in free acetic acid [2].

5-methylfurfural is associated with the deterioration of roasted coffee quality during shelf life [53]. It is associated with a sweet, caramelly, and coffee-like taste in roasted coffee, as reported by Macheiner et al. [54]. 

The percentage of this compound increased in the coffee pods for both temperatures and packaging (Table 2). After 180 days of storage, the coffee pods showed a value of 4.51% for the standard packaging and 4.76% for the alternative packaging at 25 °C. At 40 °C, there was no statistically significant difference between the standard packaging (1.01%) and alternative packaging (0.96%) for 5-methylfurfural. The coffee capsules showed a value of 2.03% at 25 °C at the 180th day in the alternative packaging and a value of 1.84% in the standard packaging, with no statistically significant difference (Table 3). For the coffee capsules at 25 °C, there was an increase of 0.49% for the standard packaging and 0.68% for the alternative packaging, while at 40 °C, for the value was 1.84% for the standard packaging and 1.40% for the alternative packaging. 

### 3.6. Sensory Evaluation

The results of the sensorial evaluation are shown in Table 4. No significant differences emerged among the samples of coffee (*p* < 0.05). In general, all coffee samples were well accepted by the consumers until 180 days of storage at 25 °C, with no differences regarding the film used for the packaging. The scores were of approximately 8, corresponding to Like very much in pods (pure Arabica), and 7, corresponding to Like very much—Like moderately in capsules (50% Arabica and Robusta blend). The higher score of the pods was related to the Arabica variety that usually has a better flavor quality than Robusta [12]. The increased values of peroxide and acetic acid during the storage did not affect the overall acceptability.

## 4. Conclusions

The metallized polyethylene terephthalate film with less aluminum used as alternative packaging had an excellent oxygen barrier performance, which is the property of utmost importance in preserving coffee freshness. The percentage of oxygen in the coffee samples packaged with this film was lower than in the samples packaged with the standard. The value did not exceed 2% during storage.

The lipidic and volatile compound trend was similar in both the alternative and standard films. Even though the peroxide values increased during 180 days of storage, they did not exceed 1.80 meq O_2_/Kg fat. The predominant volatile organic compounds detected were furans and pyrazines. Acetic acid and 5-methylfurfural increased during storage, but they did not affect overall acceptability.

Therefore, metallized polyethylene terephthalate film is an optimal choice for coffee producers because it is more sustainable than the standard, allows for reducing the packaging weight, with benefits for logistics and transportation, and preserves coffee aroma during shelf life.

## Figures and Tables

**Table 1:** Percentage (%) of moisture in coffee pods (100% *Arabica* variety) and capsules (50% *Arabica* and 50% *Robusta* varieties) packaged with standard (STD) and alternative (ALT) multilayer films and stored at 25 and 40 °C for 180 days.

Temperature		25 °C				40 °C			
Time (Days)	Time 0	90 Days		180 Days		90 Days		180 Days	
Type of Packaging		STD	ALT	STD	ALT	STD	ALT	STD	ALT
Pods	1.09 ± 0.11	1.81 ± 0.23	1.70 ± 0.15	2.15 ± 0.25	2.30 ± 0.38	1.75 ± 0.50	1.09 ± 0.11	1.81 ± 0.23	1.70 ± 0.15
Capsules	1.15 ± 0.02	1.60 ± 0.22	1.88 ± 0.13	2.59 ± 0.12	2.70 ± 0.27	2.19 ± 0.04	2.22 ± 0.33	3.25 ± 0.15	3.13 ± 0.22

No statistically significant differences were found (*p* < 0.05).

**Table 2:** Relative percentage (%) of volatile organic compounds (VOCs) in coffee pods (100% *Arabica* variety) packaged with standard (STD) and alternative (ALT) multilayer film and stored at 25 and 40 °C for 180 days.

		25 °C				40 °C			
	Time 0	90 Days		180 Days		90 Days		180 Days	
Compound		STD	ALT	STD	ALT	STD	ALT	STD	ALT
∑ Furans	36.47 ± 0.30	42.47 ± 0.06 ^b^	45.35 ± 0.08 ^a^	42.86 ± 0.15 ^b^	49.65 ± 0.06 ^a^	40.03 ± 0.10 ^b^	50.13 ± 0.15 ^a^	40.64 ± 0.20 ^b^	49.11 ± 0.20 ^a^
2-Furanmethanol	18.88 ± 0.37	20.02 ± 0.08 ^b^	25.27 ± 0.10 ^a^	24.14 ± 0.36 ^b^	29.82 ± 0.03 ^a^	19.95 ± 0.20 ^b^	25.21 ± 0.01 ^a^	23.09 ± 0.67 ^b^	29.94 ± 0.32 ^a^
Furfuryl acetate	7.28 ± 0.59	9.82 ± 0.04 ^a^	7.93 ± 0.29 ^b^	8.76 ± 0.17 ^a^	7.35 ± 0.04 ^b^	9.08 ± 0.07 ^b^	10.62 ± 0.46 ^a^	8.86 ± 0.31 ^a^	8.51 ± 0.06 ^a^
Furfuryl isovalerate	0.13 ± 0.03	0.45 ± 0.01 ^a^	0.39 ± 0.01 ^b^	0.39 ± 0.04 ^a^	0.36 ± 0.02 ^a^	0.39 ± 0.03 ^a^	0.31 ± 0.01 ^b^	0.39 ± 0.01 ^a^	0.36 ± 0.02 ^a^
Furfural	4.51 ± 0.66	4.02 ± 0.13 ^a^	3.92 ± 0.13 ^a^	2.41 ± 0.05 ^b^	5.04 ± 0.17 ^a^	4.04 ± 0.10 ^b^	5.21 ± 0.08 ^a^	2.10 ± 0.06 ^b^	2.75 ± 0.04 ^a^
Dihydro-2-methyl-3-furanone	1.49 ± 0.31	0.89 ± 0.04 ^a^	0.84 ± 0.03 ^a^	1.00 ± 0.06 ^a^	0.90 ± 0.05 ^a^	3.93 ± 0.08 ^b^	6.33 ± 0.30 ^a^	3.41 ± 0.15 ^b^	5.11 ± 0.01 ^a^
5-methyl-Furfural	3.37 ± 0.11	5.15 ± 0.01 ^b^	5.37 ± 0.01 ^a^	4.51 ± 0.14 ^a^	4.76 ± 0.06 ^a^	0.88 ± 0.03 ^a^	0.81 ± 0.02 ^a^	1.01 ± 0.05 ^a^	0.96 ± 0.01 ^a^
2,2′-Methylenebisfuran	0.53 ± 0.03	1.71 ± 0.02 ^a^	1.24 ± 0.03 ^b^	1.32 ± 0.13 ^a^	0.95 ± 0.02 ^b^	1.45 ± 0.30 ^a^	1.25 ± 0.10 ^a^	1.41 ± 0.03 ^a^	1.08 ± 0.06 ^a^
Difurfuryl ether	0.16 ± 0.04	0.31 ± 0.01 ^a^	0.30 ± 0.02 ^a^	0.24 ± 0.01 ^b^	0.36 ± 0.01 ^a^	0.22 ± 0.02 ^a^	0.28 ± 0.02 ^a^	0.21 ± 0.04 ^a^	0.29 ± 0.01 ^a^
Furfuryl methyl ether	0.12 ± 0.07	0.10 ± 0.03 ^a^	0.09 ± 0.01 ^a^	0.09 ± 0.08 ^a^	0.11 ± 0.01 ^a^	0.09 ± 0.06 ^a^	0.11 ± 0.01 ^a^	0.16 ± 0.01 ^a^	0.11 ± 0.02 ^a^
∑ Pyrazines	29.88 ± 0.34	27.46 ± 0.07 ^a^	24.41 ± 0.08 ^b^	22.04 ± 0.09 ^a^	19.89 ± 0.09 ^b^	27.36 ± 0.08 ^a^	25.12 ± 0.06 ^b^	25.20 ± 0.60 ^a^	23.30 ± 0.14 ^b^
2,5 dimethylpyrazine	17.75 ± 0.59	13.73 ± 0.05 ^a^	12.39 ± 0.03 ^b^	8.90 ± 0.39 ^a^	7.24 ± 0.40 ^b^	13.32 ± 0.31 ^a^	12.64 ± 0.06 ^a^	10.65 ± 0.39 ^a^	10.71 ± 0.17 ^a^
2-Methylpyrazine	5.47 ± 0.49	3.58 ± 0.17 ^a^	3.31 ± 0.08 ^a^	3.74 ± 0.03 ^a^	4.00 ± 0.18 ^a^	3.69 ± 0.07 ^b^	4.44 ± 0.04 ^a^	4.02 ± 0.10 ^b^	4.49 ± 0.07 ^a^
2-Ethyl-6-methylpyrazine	2.87 ± 1.20	2.48 ± 0.09 ^a^	2.22 ± 0.15 ^a^	1.62 ± 0.01 ^a^	1.85 ± 0.05 ^a^	2.68 ± 0.05 ^a^	2.35 ± 0.11 ^a^	2.44 ± 0.94 ^a^	2.30 ± 0.50 ^a^
2,3,5-Trimethylpyrazine	1.89 ± 0.04	3.05 ± 0.07 ^a^	2.41 ± 0.12 ^b^	3.14 ± 0.02 ^a^	2.56 ± 0.03 ^b^	3.31 ± 0.09 ^a^	2.25 ± 0.13 ^b^	3.44 ± 0.16 ^a^	2.27 ± 0.02 ^b^
2,5-Dimethyl-3-ethylpyrazine	0.66 ± 0.01	1.38 ± 0.01 ^a^	1.11 ± 0.01 ^b^	1.50 ± 0.01 ^a^	1.08 ± 0.02 ^b^	1.43 ± 0.06 ^a^	1.02 ± 0.07 ^b^	1.40 ± 0.06 ^a^	1.03 ± 0.08 ^b^
3-Methoxy-2-isopropylpyrazine	0.76 ± 0.15	1.48 ± 0.09 ^a^	1.33 ± 0.03 ^a^	1.51 ± 0.02 ^a^	1.20 ± 0.03 ^b^	1.16 ± 0.03 ^a^	1.04 ± 0.01 ^b^	1.11 ± 0.08 ^a^	0.96 ± 0.02 ^a^
Isopropenylpyrazine	0.22 ± 0.04	1.34 ± 0.02 ^a^	1.01 ± 0.02 ^b^	1.20 ± 0.03 ^a^	0.95 ± 0.05 ^b^	1.16 ± 0.04 ^a^	0.86 ± 0.03 ^b^	0.97 ± 0.04 ^a^	0.77 ± 0.04 ^b^
5-Methyl-6,7dihydro5-Hcyclopentapyrazine	0.15 ± 0.02	0.34 ± 0.01 ^a^	0.24 ± 0.03 ^a^	0.30 ± 0.01 ^b^	0.49 ± 0.03 ^a^	0.34 ± 0.01 ^b^	0.42 ± 0.01 ^a^	0.64 ± 0.03 ^a^	0.48 ± 0.01 ^b^
2-methyl-5-(1-propenyl) Pyrazine	0.14 ± 0.08	0.08 ± 0.02 ^b^	0.39 ± 0.01 ^a^	0.13 ± 0.05 ^b^	0.52 ± 0.05 ^a^	0.27 ± 0.03 ^a^	0.10 ± 0.01 ^b^	0.53 ± 0.04 ^a^	0.29 ± 0.01 ^b^
∑ Pyridines	10.90 ± 0.46	7.26 ± 0.12 ^a^	7.08 ± 0.09 ^a^	8.29 ± 0.08 ^a^	7.03 ± 0.03 ^b^	8.21 ± 0.05 ^a^	3.81 ± 0.03 ^b^	7.76 ± 0.06 ^a^	3.97 ± 0.01 ^b^
Pyridine	9.67 ± 0.33	4.85 ± 0.36 ^a^	5.24 ± 0.20 ^a^	5.78 ± 0.18 ^a^	5.90 ± 0.06 ^a^	5.96 ± 0.10 ^a^	3.29 ± 0.07 ^b^	5.79 ± 0.12 ^a^	3.71 ± 0.01 ^b^
1-Methyl-1,2,3,6-tetrahydropyridine	0.88 ± 0.12	2.06 ± 0.01 ^a^	1.54 ± 0.04 ^b^	2.17 ± 0.02 ^a^	0.86 ± 0.03 ^b^	1.89 ± 0.06 ^a^	0.37 ± 0.01 ^b^	1.63 ± 0.09 ^a^	0.13 ± 0.01 ^b^
3-Ethylpyridine	0.36 ± 0.01	0.35 ± 0.03 ^a^	0.30 ± 0.02 ^a^	0.34 ± 0.04 ^a^	0.27 ± 0.01 ^b^	0.36 ± 0.03 ^a^	0.15 ± 0.01 ^b^	0.34 ± 0.04 ^a^	0.13 ± 0.01 ^b^
∑ Ketones	11.03 ± 1.06	6.81 ± 0.02 ^b^	7.01 ± 0.06 ^a^	8.25 ± 0.03 ^a^	6.88 ± 0.03 ^b^	6.03 ± 0.05 ^a^	4.60 ± 0.05 ^b^	7.02 ± 0.04 ^a^	5.56 ± 0.04 ^b^
1-(Acetyloxy)-2-propanone	8.27 ± 0.13	4.30 ± 0.03 ^a^	3.99 ± 0.21 ^a^	4.23 ± 0.05 ^a^	4.11 ± 0.05 ^b^	3.42 ± 0.18 ^a^	2.82 ± 0.15 ^b^	3.83 ± 0.06 ^a^	3.35 ± 0.08 ^b^
Acetone	1.42 ± 1.07	0.36 ± 0.04 ^b^	1.07 ± 0.02 ^a^	1.87 ± 0.01 ^a^	1.29 ± 0.04 ^b^	1.03 ± 0.04 ^a^	0.65 ± 0.03 ^b^	1.66 ± 0.10 ^a^	0.99 ± 0.04 ^b^
3-Hydroxy-2-butanone	0.74 ± 0.13	0.65 ± 0.01 ^a^	0.45 ± 0.01 ^b^	0.63 ± 0.01 ^a^	0.44 ± 0.01 ^b^	0.53 ± 0.01 ^a^	0.31 ± 0.02 ^b^	0.59 ± 0.04 ^a^	0.32 ± 0.01 ^b^
2-Hydroxy-3-methyl-2-cyclopenten-1-one	0.34 ± 0.04	0.80 ± 0.01 ^a^	0.86 ± 0.04 ^a^	0.89 ± 0.02 ^a^	0.57 ± 0.02 ^b^	0.50 ± 0.01 ^a^	0.45 ± 0.02 ^a^	0.48 ± 0.04 ^a^	0.54 ± 0.02 ^a^
3-Ethyl-2-hydroxy-2-cyclopentenone	0.27 ± 0.06	0.70 ± 0.01 ^a^	0.64 ± 0.02 ^a^	0.63 ± 0.01 ^a^	0.47 ± 0.01 ^b^	0.55 ± 0.02 ^a^	0.37 ± 0.01 ^b^	0.46 ± 0.02 ^a^	0.36 ± 0.01 ^b^
∑Phenols	1.61 ± 0.04	4.09 ± 0.02 ^a^	3.57 ± 0.06 ^b^	3.41 ± 0.03 ^a^	2.59 ± 0.03 ^b^	2.97 ± 0.07 ^a^	2.89 ± 0.08 ^a^	2.64 ± 0.03 ^a^	2.46 ± 0.04 ^b^
Guaiacol	0.74 ± 0.07	1.00 ± 0.01 ^b^	1.20 ± 0.16 ^a^	0.86 ± 0.05 ^a^	0.71 ± 0.02 ^b^	0.71 ± 0.21 ^a^	0.86 ± 0.27 ^a^	0.52 ± 0.02 ^a^	0.68 ± 0.02 ^b^
4-Vinylphenol	0.42 ± 0.04	1.94 ± 0.04 ^a^	1.37 ± 0.02 ^b^	1.55 ± 0.01 ^a^	1.04 ± 0.03 ^b^	1.36 ± 0.02 ^a^	1.03 ± 0.02 ^b^	1.07 ± 0.04 ^a^	0.77 ± 0.03 ^b^
4-ethyl-2-methoxyphenol	0.30 ± 0.02	0.82 ± 0.01 ^a^	0.75 ± 0.05 ^a^	0.72 ± 0.01 ^a^	0.49 ± 0.02 ^b^	0.74 ± 0.05 ^a^	0.63 ± 0.07 ^a^	0.77 ± 0.03 ^a^	0.59 ± 0.01 ^b^
Phenol	0.15 ± 0.03	0.33 ± 0.02 ^a^	0.25 ± 0.02 ^b^	0.28 ± 0.02 ^a^	0.35 ± 0.04 ^a^	0.16 ± 0.03 ^b^	0.37 ± 0.05 ^a^	0.28 ± 0.02 ^b^	0.42 ± 0.06 ^a^
∑Pyrroles	1.82 ± 0.30	3.72 ± 0.03 ^a^	3.15 ± 0.03 ^b^	4.03 ± 0.04 ^a^	3.73 ± 0.02 ^b^	4.30 ± 0.03 ^a^	3.33 ± 0.03 ^b^	4.15 ± 0.05 ^a^	4.04 ± 0.04 ^a^
1-Furfurylpyrrole	0.76 ± 0.01	0.98 ± 0.04 ^a^	0.76 ± 0.03 ^b^	1.70 ± 0.08 ^a^	1.34 ± 0.01 ^b^	1.58 ± 0.01 ^a^	0.79 ± 0.02 ^b^	1.56 ± 0.06 ^a^	1.46 ± 0.04 ^a^
2-Acetyl-1-methylpyrrole	0.75 ± 0.24	1.47 ± 0.05 ^a^	1.18 ± 0.05 ^b^	1.33 ± 0.03 ^a^	1.07 ± 0.01 ^b^	1.59 ± 0.03 ^a^	1.38 ± 0.03 ^b^	1.61 ± 0.06 ^a^	1.27 ± 0.03 ^b^
2-Acetylpyrrole	0.31 ± 0.06	0.99 ± 0.02 ^a^	0.99 ± 0.03 ^a^	0.77 ± 0.02 ^b^	1.09 ± 0.02 ^a^	0.94 ± 0.01 ^a^	1.01 ± 0.02 ^a^	0.81 ± 0.06 ^b^	1.12 ± 0.05 ^a^
1H-pyrrole-2-carboxaldehyde	n.d.	0.28 ± 0.02 ^a^	0.22 ± 0.01 ^b^	0.23 ± 0.01 ^a^	0.23 ± 0.02 ^a^	0.19 ± 0.01 ^a^	0.15 ± 0.03 ^a^	0.17 ± 0.02 ^a^	0.19 ± 0.04 ^a^
∑ Aldehydes	0.76 ± 0.29	1.68 ± 0.03 ^a^	1.20 ± 0.02 ^b^	1.82 ± 0.02 ^a^	1.05 ± 0.03 ^b^	1.72 ± 0.01 ^a^	0.88 ± 0.01 ^b^	1.92 ± 0.05 ^a^	0.79 ± 0.02 ^b^
2-Butenal	0.45 ± 0.06	1.19 ± 0.06 ^a^	1.20 ± 0.02 ^a^	1.24 ± 0.01 ^a^	1.05 ± 0.03 ^b^	1.27 ± 0.01 ^a^	0.88 ± 0.01 ^b^	1.13 ± 0.06 ^a^	0.79 ± 0.02 ^b^
Benzaldehyde	0.31 ± 0.23	0.49 ± 0.01 ^b^	n.d.	0.58 ± 0.02 ^a^	n.d.	0.45 ± 0.01 ^a^	n.d.	0.79 ± 0.04 ^a^	n.d.
∑ Others	7.58 ± 0.39	6.52 ± 0.02 ^b^	8.23 ± 0.07 ^a^	9.30 ± 0.09 ^a^	9.18 ± 0.04 ^a^	9.38 ± 0.07 ^a^	9.24 ± 0.14 ^a^	10.66 ± 0.10 ^a^	10.77 ± 0.03 ^a^
Acetic acid	7.11 ± 0.34	5.26 ± 0.04 ^b^	6.98 ± 0.25 ^a^	8.24 ± 0.31 ^a^	8.50 ± 0.08 ^a^	8.73 ± 0.20 ^a^	8.87 ± 0.46 ^a^	10.11 ± 0.35 ^a^	10.27 ± 0.05 ^a^
Maltol	0.43 ± 0.07	1.21 ± 0.02 ^a^	1.21 ± 0.01 ^a^	0.99 ± 0.01 ^a^	0.63 ± 0.01 ^b^	0.61 ± 0.02 ^a^	0.32 ± 0.06 ^b^	0.50 ± 0.02 ^a^	0.44 ± 0.05 ^a^
3,4-Dimethoxystyrene	0.04 ± 0.05	0.05 ± 0.03 ^a^	0.04 ± 0.02 ^b^	0.07 ± 0.03 ^a^	0.05 ± 0.01 ^a^	0.04 ± 0.03 ^a^	0.05 ± 0.01 ^a^	0.05 ± 0.02 ^a^	0.06 ± 0.01 ^a^
4-Ethyl-decane	n.d.	n.d.	n.d.	n.d.	n.d.	n.d.	n.d.	n.d.	n.d.

^a–b^ Different letters at the same time and temperature indicate statistically significant differences (*p* < 0.05); n.d. not detected.

**Table 3:** Relative percentage (%) of volatile organic compounds (VOCs) in coffee capsules (50% *Arabica* and 50% *Robusta* varieties) packaged with standard (STD) and alternative (ALT) multilayer film and stored at 25 and 40 °C for 180 days.

		25 °C				40 °C			
	Time 0	90 Days		180 Days		90 Days		180 Days	
Compound		STD	ALT	STD	ALT	STD	ALT	STD	ALT
∑ Furans	27.40 ± 0.30	37.3 ± 0.09 ^a^	36.11 ± 0.14 ^a^	34.65 ± 0.07 ^b^	34.69 ± 0.09 ^a^	36.69 ± 0.04 ^b^	42.91 ± 0.10 ^a^	34.65 ± 0.07 ^b^	44.06 ± 0.03 ^a^
2-Furanmethanol	11.68 ± 2.28	19.13 ± 0.04 ^a^	17.57 ± 0.30 ^b^	16.34 ± 0.06 ^a^	20.26 ± 0.07 ^b^	13.53 ± 0.06 ^b^	22.49 ± 0.28 ^a^	16.34 ± 0.06 ^b^	24.10 ± 0.03 ^a^
Furfuryl acetate	6.44 ± 1.81	8.74 ± 0.15 ^b^	9.24 ± 0.05 ^a^	6.84 ± 0.13 ^a^	5.18 ± 0.25 ^b^	7.99 ± 0.02 ^a^	5.95 ± 0.06 ^b^	6.84 ± 0.13 ^a^	6.76 ± 0.04 ^b^
Furfuryl isovalerate	1.15 ± 0.18	2.90 ± 0.05 ^a^	1.91 ± 0.04 ^b^	3.99 ± 0.12 ^a^	2.71 ± 0.06 ^b^	9.59 ± 0.01 ^a^	6.34 ± 0.03 ^b^	3.99 ± 0.12 ^b^	5.72 ± 0.04 ^a^
Furfural	3.36 ± 0.17	1.21 ± 0.09 ^b^	2.50 ± 0.07 ^a^	2.53 ± 0.16 ^a^	1.28 ± 0.06 ^b^	1.96 ± 0.03 ^b^	3.00 ± 0.17 ^a^	2.53 ± 0.16 ^b^	3.24 ± 0.06 ^a^
Dihydro-2-methyl-3-furanone	2.66 ± 0.84	2.58 ± 0.08 ^a^	2.11 ± 0.06 ^b^	2.06 ± 0.09 ^a^	2.07 ± 0.08 ^b^	2.20 ± 0.08 ^a^	2.20 ± 0.01 ^a^	2.06 ± 0.09 ^a^	1.97 ± 0.02 ^a^
5-methyl-Furfural	1.35 ± 0.28	1.62 ± 0.02 ^a^	1.78 ± 0.08 ^a^	1.84 ± 0.14 ^b^	2.03 ± 0.10 ^a^	1.94 ± 0.06 ^a^	1.48 ± 0.09 ^b^	1.84 ± 0.14 ^a^	1.40 ± 0.05 ^b^
2,2′-Methylenebisfuran	0.29 ± 0.12	0.47 ± 0.01 ^a^	0.50 ± 0.01 ^a^	0.45 ± 0.01 ^b^	0.50 ± 0.01 ^a^	0.98 ± 0.02 ^a^	0.60 ± 0.01 ^b^	0.45 ± 0.01 ^a^	0.50 ± 0.02 ^a^
Difurfuryl ether	0.32 ± 0.06	0.56 ± 0.01 ^a^	0.34 ± 0.03 ^b^	0.46 ± 0.04 ^a^	0.30 ± 0.01 ^b^	0.31 ± 0.01 ^a^	0.32 ± 0.02 ^a^	0.46 ± 0.04 ^a^	0.25 ± 0.03 ^b^
Furfuryl methyl ether	0.15 ± 0.05	0.09 ± 0.01 ^b^	0.16 ± 0.01 ^a^	0.14 ± 0.02 ^a^	0.16 ± 0.02 ^a^	0.19 ± 0.02 ^b^	0.50 ± 0.01 ^a^	0.14 ± 0.02 ^a^	0.12 ± 0.01 ^a^
∑ Pyrazines	34.22 ± 0.23	28.62 ± 0.22 ^b^	32.42 ± 0.09 ^a^	31.84 ± 0.18 ^a^	30.38 ± 0.11 ^b^	33.37 ± 0.11 ^a^	29.28 ± 0.04 ^b^	31.84 ± 0.18 ^a^	27.11 ± 0.03 ^b^
2,5 dimethylpyrazine	15.08 ± 0.86	9.49 ± 0.03 ^b^	14.66 ± 0.10 ^a^	13.91 ± 0.16 ^a^	13.02 ± 0.47 ^a^	14.65 ± 0.45 ^a^	13.14 ± 0.03 ^b^	13.91 ± 0.16 ^a^	10.86 ± 0.01 ^b^
2-Methylpyrazine	6.08 ± 1.45	2.42 ± 0.15 ^b^	3.59 ± 0.05 ^a^	1.89 ± 0.05 ^b^	3.21 ± 0.02 ^a^	1.97 ± 0.07 ^a^	1.38 ± 0.02 ^b^	1.89 ± 0.05 ^a^	1.62 ± 0.03 ^b^
2-Ethyl-6-methylpyrazine	4.35 ± 0.60	7.86 ± 0.38 ^a^	6.60 ± 0.11 ^b^	7.62 ± 0.24 ^a^	7.14 ± 0.17 ^a^	8.00 ± 0.08 ^a^	7.50 ± 0.12 ^b^	7.62 ± 0.24 ^a^	7.19 ± 0.02 ^b^
2,3,5-Trimethylpyrazine	4.68 ± 1.34	2.94 ± 0.06 ^a^	2.19 ± 0.08 ^b^	2.71 ± 0.17 ^a^	2.01 ± 0.03 ^b^	2.79 ± 0.01 ^a^	1.96 ± 0.08 ^b^	2.71 ± 0.17 ^a^	2.93 ± 0.07 ^b^
2,5-Dimethyl-3-ethylpyrazine	1.90 ± 0.31	2.61 ± 0.02 ^a^	2.26 ± 0.02 ^b^	2.51 ± 0.05 ^a^	2.16 ± 0.03 ^b^	2.66 ± 0.08 ^a^	1.71 ± 0.03 ^b^	2.51 ± 0.05 ^a^	1.80 ± 0.04 ^b^
3-Methoxy-2-isopropylpyrazine	0.91 ± 0.11	1.32 ± 0.06 ^a^	1.05 ± 0.03 ^b^	1.16 ± 0.03 ^a^	0.90 ± 0.09 ^a^	1.36 ± 0.04 ^b^	1.77 ± 0.01 ^a^	1.16 ± 0.03 ^a^	1.16 ± 0.01 ^a^
Isopropenylpyrazine	0.41 ± 0.18	0.31 ± 0.04 ^b^	0.55 ± 0.02 ^a^	0.36 ± 0.02 ^b^	0.51 ± 0.03 ^a^	0.39 ± 0.02 ^a^	0.44 ± 0.05 ^a^	0.36 ± 0.02 ^a^	0.23 ± 0.01 ^a^
5-Methyl-6.7dihydro5-Hcyclopentapyrazine	0.46 ± 0.06	1.28 ± 0.08 ^a^	1.19 ± 0.02 ^a^	1.29 ± 0.03 ^a^	1.08 ± 0.04 ^b^	1.14 ± 0.02 ^a^	1.15 ± 0.03 ^a^	1.29 ± 0.03 ^a^	1.05 ± 0.04 ^b^
2-methyl-5-(1-propenyl) Pyrazine	0.35 ± 0.05	0.39 ± 0.01 ^a^	0.33 ± 0.02 ^b^	0.39 ± 0.03 ^a^	0.35 ± 0.02 ^a^	0.41 ± 0.04 ^a^	0.23 ± 0.02 ^b^	0.39 ± 0.03 ^a^	0.27 ± 0.03 ^b^
∑Pyridines	14.29 ± 1.52	10.87 ± 0.04 ^a^	9.92 ± 0.08 ^b^	10.89 ± 0.15 ^b^	11.32 ± 0.05 ^a^	9.71 ± 0.07 ^a^	8.96 ± 0.04 ^b^	10.89 ± 0.15 ^a^	8.28 ± 0.02 ^b^
Pyridine	13.03 ± 1.18	8.32 ± 0.06 ^a^	7.63 ± 0.02 ^b^	8.44 ± 0.24 ^b^	8.96 ± 0.07 ^a^	7.59 ± 0.15 ^a^	7.68 ± 0.07 ^a^	8.44 ± 0.24 ^a^	7.84 ± 0.03 ^b^
1-Methyl-1.2.3.6-tetrahydropyridine	0.97 ± 0.11	1.98 ± 0.06 ^a^	1.77 ± 0.11 ^b^	1.89 ± 0.04 ^a^	1.93 ± 0.02 ^a^	1.49 ± 0.01 ^a^	0.83 ± 0.03 ^b^	1.89 ± 0.04 ^a^	0.16 ± 0.01 ^b^
3-Ethylpyridine	0.30 ± 0.03	0.57 ± 0.01 ^a^	0.52 ± 0.02 ^a^	0.56 ± 0.02 ^a^	0.43 ± 0.02 ^b^	0.63 ± 0.04 ^a^	0.45 ± 0.01 ^b^	0.56 ± 0.02 ^a^	0.28 ± 0.03 ^b^
∑ Ketones	7.30 ± 0.35	6.33 ± 0.07 ^a^	5.91 ± 0.05 ^b^	5.69 ± 0.08 ^a^	5.78 ± 0.08 ^a^	4.20 ± 0.02 ^b^	4.88 ± 0.04 ^a^	5.69 ± 0.08 ^a^	3.57 ± 0.02 ^b^
1-(Acetyloxy)-2-propanone	5.20 ± 0.06	3.73 ± 0.19 ^a^	3.43 ± 0.09 ^a^	3.40 ± 0.22 ^a^	3.32 ± 0.17 ^a^	2.72 ± 0.02 ^a^	2.84 ± 0.07 ^a^	3.40 ± 0.22 ^a^	2.19 ± 0.01 ^b^
Acetone	1.04 ± 0.14	0.98 ± 0.04 ^a^	0.96 ± 0.03 ^a^	1.06 ± 0.05 ^a^	0.99 ± 0.16 ^a^	0.78 ± 0.03 ^b^	1.09 ± 0.09 ^a^	1.06 ± 0.05 ^a^	0.73 ± 0.04 ^b^
3-Hydroxy-2-butanone	0.47 ± 0.13	0.63 ± 0.05 ^a^	0.60 ± 0.02 ^a^	0.46 ± 0.02 ^b^	0.69 ± 0.05 ^a^	0.32 ± 0.03 ^a^	0.37 ± 0.02 ^a^	0.46 ± 0.02 ^a^	0.19 ± 0.01 ^b^
2-Hydroxy-3-methyl-2-cyclopenten-1-one	0.40 ± 0.01	0.61 ± 0.05 ^a^	0.57 ± 0.03 ^a^	0.47 ± 0.03 ^a^	0.54 ± 0.03 ^a^	0.38 ± 0.02 ^a^	0.38 ± 0.01 ^a^	0.47 ± 0.03 ^a^	0.26 ± 0.02 ^b^
3-Ethyl-2-hydroxy-2-cyclopentenone	0.19 ± 0.01	0.38 ± 0.01 ^a^	0.35 ± 0.02 ^a^	0.30 ± 0.01 ^a^	0.24 ± 0.01 ^b^	n.d.	0.20 ± 0.04 ^a^	0.30 ± 0.01 ^a^	0.20 ± 0.02 ^b^
∑ Phenols	5.46 ± 1.68	8.03 ± 0.07 ^a^	7.71 ± 0.02 ^b^	8.27 ± 0.05 ^a^	7.29 ± 0.04 ^b^	4.52 ± 0.02 ^a^	4.01 ± 0.03 ^b^	8.27 ± 0.05 ^a^	3.83 ± 0.01 ^b^
Guaiacol	2.28 ± 0.05	2.62 ± 0.12 ^a^	2.54 ± 0.02 ^a^	2.16 ± 0.06 ^a^	2.21 ± 0.06 ^a^	1.91 ± 0.03 ^a^	1.97 ± 0.06 ^a^	2.16 ± 0.06 ^a^	1.88 ± 0.02 ^b^
4-Vinylphenol	1.47 ± 0.03	2.89 ± 0.07 ^a^	2.86 ± 0.02 ^a^	3.90 ± 0.02 ^a^	3.40 ± 0.06 ^b^	0.76 ± 0.02 ^a^	0.47 ± 0.03 ^b^	3.90 ± 0.02 ^a^	0.59 ± 0.01 ^b^
4-ethyl-2-methoxyphenol	0.95 ± 0.21	1.91 ± 0.05 ^a^	1.69 ± 0.01 ^b^	1.87 ± 0.07 ^a^	1.20 ± 0.03 ^b^	1.50 ± 0.04 ^a^	1.05 ± 0.01 ^b^	1.87 ± 0.07 ^a^	0.83 ± 0.01 ^b^
Phenol	0.77 ± 0.19	0.61 ± 0.06 ^a^	0.62 ± 0.02 ^a^	0.34 ± 0.03^cb^	0.48 ± 0.02 ^a^	0.35 ± 0.01 ^b^	0.52 ± 0.01 ^a^	0.34 ± 0.03 ^b^	0.53 ± 0.01 ^a^
∑ Pyrroles	3.13 ± 0.16	3.17 ± 0.02 ^a^	2.71 ± 0.02 ^b^	3.05 ± 0.04 ^a^	2.96 ± 0.04 ^b^	3.18 ± 0.02 ^a^	3.06 ± 0.02 ^a^	3.05 ± 0.04 ^b^	3.23 ± 0.03 ^a^
1-Furfurylpyrrole	0.97 ± 0.34	0.70 ± 0.01 ^a^	0.73 ± 0.01 ^a^	0.61 ± 0.02 ^b^	0.90 ± 0.04 ^a^	0.71 ± 0.01 ^a^	0.44 ± 0.02 ^b^	0.61 ± 0.02 ^b^	0.83 ± 0.04 ^a^
2-Acetyl-1-methylpyrrole	1.49 ± 0.54	1.47 ± 0.03 ^a^	0.86 ± 0.02 ^b^	1.56 ± 0.04 ^a^	1.27 ± 0.04 ^b^	1.50 ± 0.04 ^b^	1.81 ± 0.04 ^a^	1.56 ± 0.04 ^b^	1.75 ± 0.07 ^a^
2-Acetylpyrrole	0.57 ± 0.01	0.91 ± 0.03 ^a^	0.84 ± 0.02 ^a^	0.79 ± 0.07 ^a^	0.67 ± 0.04 ^b^	0.87 ± 0.05 ^a^	0.72 ± 0.04 ^b^	0.79 ± 0.07 ^a^	0.57 ± 0.04 ^b^
1H-pyrrole-2-carboxaldehyde	0.10 ± 0.02	0.09 ± 0.01 ^b^	0.28 ± 0.01 ^a^	0.09 ± 0.02 ^b^	0.12 ± 0.03 ^b^	0.10 ± 0.05 ^a^	0.09 ± 0.01 ^a^	0.09 ± 0.02 ^a^	0.08 ± 0.07 ^a^
∑ Aldehydes	0.61 ± 0.02	0.95 ± 0.03 ^b^	1.12 ± 0.03 ^a^	1.08 ± 0.01 ^a^	1.00 ± 0.02 ^b^	0.93 ± 0.02 ^a^	0.49 ± 0.01 ^b^	1.08 ± 0.01 ^a^	0.66 ± 0.02 ^b^
2-Butenal	0.36 ± 0.10	0.45 ± 0.02 ^b^	0.71 ± 0.02 ^a^	0.68 ± 0.01 ^a^	0.57 ± 0.04 ^b^	0.60 ± 0.04 ^a^	0.20 ± 0.01 ^b^	0.68 ± 0.01 ^a^	0.34 ± 0.02 ^b^
Benzaldehyde	0.25 ± 0.02	0.50 ± 0.07 ^a^	0.41 ± 0.03 ^b^	0.40 ± 0.01 ^a^	0.43 ± 0.01 ^a^	0.33 ± 0.01 ^a^	0.29 ± 0.01 ^a^	0.40 ± 0.01 ^a^	0.32 ± 0.01 ^b^
∑ Others	7.59 ± 2.98	4.73 ± 0.07 ^a^	4.10 ± 0.06 ^b^	4.52 ± 0.17 ^b^	6.57 ± 0.04 ^a^	5.38 ± 0.04 ^a^	6.46 ± 0.04 ^b^	4.52 ± 0.17 ^b^	9.27 ± 0.03 ^a^
Acetic acid	6.37 ± 1.60	3.09 ± 0.13 ^a^	3.05 ± 0.08 ^a^	3.71 ± 0.29 ^b^	5.84 ± 0.09 ^a^	4.69 ± 0.08 ^b^	5.82 ± 0.05 ^a^	3.71 ± 0.29 ^b^	8.83 ± 0.05 ^a^
Maltol	0.70 ± 0.20	0.66 ± 0.05 ^a^	0.53 ± 0.03 ^b^	0.42 ± 0.01 ^a^	0.39 ± 0.05 ^a^	0.23 ± 0.02 ^b^	0.36 ± 0.02 ^a^	0.42 ± 0.01 ^a^	0.15 ± 0.04 ^b^
3.4-Dimethoxystyrene	0.36 ± 0.02	0.69 ± 0.02 ^a^	0.24 ± 0.01 ^b^	0.12 ± 0.05 ^a^	0.13 ± 0.01 ^a^	0.19 ± 0.03 ^a^	0.10 ± 0.02 ^b^	0.12 ± 0.05 ^a^	0.11 ± 0.03 ^a^
4-Ethyl-decano	0.16 ± 0.09	0.29 ± 0.01 ^a^	0.28 ± 0.01 ^a^	0.27 ± 0.01 ^a^	0.21 ± 0.02 ^b^	0.27 ± 0.03 ^a^	0.18 ± 0.01 ^b^	0.27 ± 0.01 ^a^	0.18 ± 0.01^c^

^a–c^ Different letters at the same time and temperature indicate statistically significant differences (*p* < 0.05); n.d. not detected.

**Table 4:** Overall acceptability of coffee from pods (100% *Arabica* variety) and capsules (50% *Arabica* and 50% *Robusta* varieties) packaged with standard (STD) and alternative (ALT) multilayer film and stored at 25 °C for 180 days.

Temperature		25 °C			
Time (Days)	Time 0	90 Days		180 Days	
Type of Packaging		STD	ALT	STD	ALT
Pods	8.50 ^a^ ± 0.50	8.50 ^a^ ± 1.00	8.50 ^a^ ± 0.50	8.00 ^a^ ± 1.00	8.00 ^a^ ± 0.50
Capsules	7.50 ^b^ ± 0.50	7.50 ^b^ ± 0.50	7.50 ^b^ ± 0.50	7.00 ^b^ ± 0.50	7.00 ^b^ ± 0.50

No statistically significant differences were found (*p* < 0.05).

## Data Availability

The original contributions presented in the study are included in the article, further inquiries can be directed to the corresponding author.
